# Molecular Fingerprints of Malignant Pleural Mesothelioma: Not Just a Matter of Genetic Alterations

**DOI:** 10.3390/jcm10112470

**Published:** 2021-06-02

**Authors:** Eugenia Lorenzini, Alessia Ciarrocchi, Federica Torricelli

**Affiliations:** 1 Laboratory of Translational Research, Azienda USL—IRCCS di Reggio Emilia, 42123 Reggio Emilia, Italy; eugenia.lorenzini@ausl.re.it (E.L.); alessia.ciarrocchi@ausl.re.it (A.C.); 2 Department of Pharmacy and Biotechnology (FABIT), University of Bologna, 40126 Bologna, Italy

**Keywords:** malignant pleural mesothelioma, epigenome, target therapies

## Abstract

Malignant pleural mesothelioma (MPM) is a clinical emergency of our time. Being strongly associated with asbestos exposure, incidence of this cancer is ramping up these days in many industrialized countries and it will soon start to increase in many developing areas where the use of this silicate derivate is still largely in use. Deficiency of reliable markers for the early identification of these tumors and the limited efficacy of the currently available therapeutic options are the basis of the impressive mortality rate of MPM. These shortcomings reflect the very poor information available about the molecular basis of this disease. Results of the recently released deep profiling studies point to the epigenome as a central element in MPM development and progression. First, MPM is characterized by a low mutational burden and a highly peculiar set of mutations that hits almost exclusively epigenetic keepers or proteins controlling chromatin organization and function. Furthermore, asbestos does not seem to be associated with a distinctive mutational signature, while the precise mapping of epigenetic changes caused by this carcinogen has been defined, suggesting that alterations in epigenetic features are the driving force in the development of this disease. Last but not least, consistent evidence also indicates that, in the setting of MPM, chromatin rewiring and epigenetic alterations of cancer cells heavily condition the microenvironment, including the immune response. In this review we aim to point to the relevance of the epigenome in MPM and to highlight the dependency of this tumor on chromatin organization and function. We also intend to discuss the opportunity of targeting these mechanisms as potential therapeutic options for MPM.

## 1. Introduction

Malignant pleural mesothelioma (MPM) is a very rare but aggressive cancer arising from the mesothelial cells lining the lungs’ pleura [1,2,3,4,5].

It is characterized by a high mortality rate and a dismal prognosis due to limited treatment options available. Over 80% of MPMs are associated with professional or environmental chronic asbestos exposure, even if difficulties in tracking this contaminant and the long latency period between the exposure and tumor development make this percentage largely underestimated. Despite asbestos restrictions, incidence and mortality rates for this cancer are expected to rise in the next decade in many industrialized countries. As well, a considerable increase of MPM cases is predicted in emerging economies, including China and India, where asbestos is still largely used [6].

Morphologically, three main MPM histotypes can be recognized, reflecting the degree of cell differentiation, that also mirror different clinical behaviors: epithelioid, sarcomatoid, and biphasic MPM [5,7], with the sarcomatoid subtype being the most aggressive and the one with the worst prognosis [8].

The molecular basis of this disease remains largely unknown, currently representing the most relevant limitation to the development of effective MPM targeting strategies [9].

Insights into the genetic landscape of this tumor by recent deep sequencing studies revealed a quite low mutational burden and only few pathologically important mutations. Furthermore, no specific genetic changes that can be attributed to the action of asbestos could be identified [8,10,11].

Instead, increasing evidence points to the epigenome as a primary target of asbestos and as a central hub in the genesis and progression of mesothelioma. An extremely large number of loci, many of which are implicated in cell cycle regulation, have been shown to be epigenetically altered in MPM and to correlate with asbestos exposure. Logically, with an epigenome-based evolution of MPM its decades-long latency would allow ample time for cellular turnover and selection of cells with altered epigenetic programs, thus favoring survival and deregulated proliferation.

Chromatin plasticity is at the basis of many fundamental processes like transcription, chromosome condensation, and DNA Damage Repair (DDR). All these processes are busted in cancer to keep up with the massive proliferation capacity of tumor cells. Furthermore, consistent evidence has demonstrated that chromatin rewiring may heavily condition the cancer microenvironment, including immune response [11,12,13].

In this review we aim to point to the relevance of the epigenome in MPM and the dependency of this tumor on chromatin organization and function.

Literature from the very early days of mesothelioma research was considered and included, as well as the most recent data published at the beginning of 2021, resulting in 121 references.

## 2. Asbestos-Mediated Epigenetic Changes

The majority of MPM cases are a consequence of chronic asbestos exposure. Asbestos consists of a group of naturally occurring silicate minerals that were largely used in the sectors of industry and construction until a few decades ago [14]. Asbestos fibers are associated with the development of both malignant (lung cancers, mesothelioma) and non-malignant (asbestosis) diseases [15]. Noticeably, while asbestos is known to cause genotoxicity through DNA breaks and oxidative damage, no association between asbestos exposure and a precise and recurrent MPM mutational profile could be established, thus failing to identify an asbestos-associated mutational signature at the origin of MPM [8,11,16].

By contrast, evidence indicates that asbestos is associated with epigenetic changes even if the molecular mechanisms through which this occurs are not fully understood. Inhalation of asbestos fibers results in their deposition in the lungs, causing a profound inflammatory response that involves the continuous production of free radicals, reactive oxygen (ROS) and nitrogen (RNS) species [3,17,18]. ROS and RNS collide with cellular components, promoting DNA mutation and triggering transformation [14,19]. Moreover, the majority of asbestos fibers contain iron. Iron ions (Fe^2+^) are able to induce hemolysis, sequestering iron from hemoglobin and releasing it [14]. Free iron is the catalyst of the Fenton reaction that results in the generation of hydroxyl radicals (OH). These radicals can oxidate DNA, proteins, and other biological molecules, damaging them [20]. In particular, OH can hydroxylate the C8 position of deoxyguanosine, forming 8-hydroxy-2′-deoxyguanosine (8-OHdG), thus generating DNA base mispairing and G-to-T transversions [21].

The inflammatory process is exacerbated by the activation of macrophages that are stimulated, among others, by the release of high-mobility group box 1 protein (HMGB1) as well as the secretion of TNF-alpha and other inflammatory cytokines in the intercellular spaces.

These pro-inflammatory molecules converge upon the activation of NF-Kb, which helps human mesenchymal-damaged cells to survive and thus promotes tumor establishment and progression [22,23].

**Figure 1:**
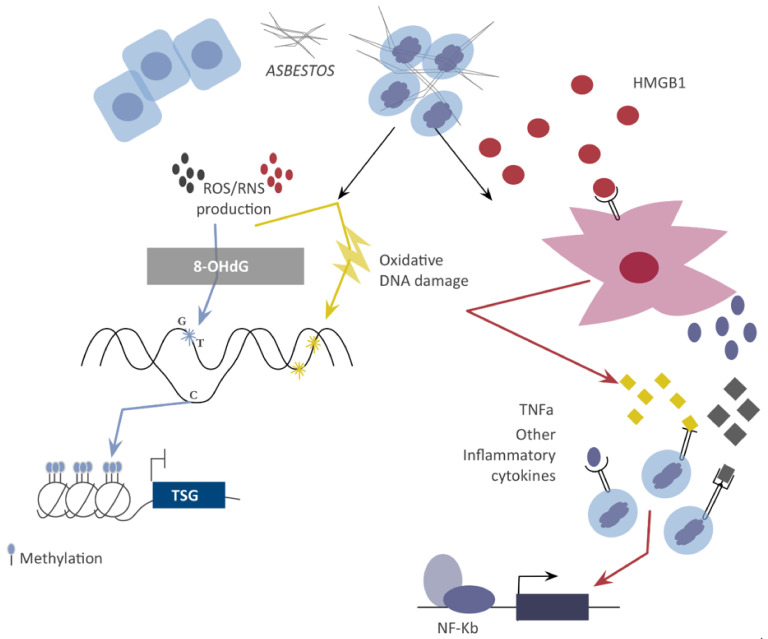
Inhalation of asbestos fibers causes an inflammatory response in the lungs that results in the chronic production of ROS and RNS. These reactive species collide with biological molecules, damaging them. In particular, 8-OhdG can generate DNA base mispairing, resulting in G-to-T transversions. Moreover, DNA methylation is vulnerable to asbestos fibers that, indeed, are a cause of tumor suppressor genes (TSGs), promoting hypermethylation and subsequent silencing. Additionally, mesothelial cells exposed to asbestos undergo necrosis, releasing HMGB1 into the intercellular space and thus recruiting macrophages and stimulating the chronic inflammation response. Activated macrophages release TNF-alpha and other inflammatory cytokines that result in NF-kB. Activation leads to consequent survival of HM cells with genetic damage. These two mechanisms work together to trigger tumor formation and growth. List of abbreviations: ROS—reactive oxygen species, RNS—reactive nitrogen species, TSGs—tumor suppressor genes, HMGB1—high-mobility group box 1, TNFa—tumor necrosis factor alpha.

While acute production of ROS generates largely reparable DNA mutations, exposure to chronic ROS production, like the one imposed by the asbestos fibers, produces a dangerous stressed microenvironment in which consequences are harder to overcome [24] (Figure 1).

This chronic inflammation caused by the exposure to asbestos also impacts the overall epigenetic landscape of HM cells, in particular via the alteration of their methylation profile. Relevant evidence supports an association of asbestos and chronic inflammation with hypermethylation of crucial oncosuppressor genes [25] with an impact also on clinical outcomes of MPM patients [26]. Moreover, asbestos exposure seems to be involved in microRNA expression. Alterations in these molecules induce epigenetic changes favoring MPM development and progression (see below) [27,28,29,30].

## 3. The Genomic Landscape of MPM Is Characterized by Alterations in Epigenetic Keepers

Deep analysis of the genomic landscape of MPM showed that this tumor is characterized by a low mutational burden with a mean of <2 somatic non-synonymous mutations per megabase and few recurrent gene mutations [11]. Furthermore, studies have highlighted that MPM is characterized by a quite peculiar set of alterations hitting in particular proteins involved in chromatin organization and epigenetic keepers.

*BAP1* is a deubiquitinating enzyme that binds to the breast cancer type 1 susceptibility protein (BRCA1) via the RING finger domain, and through this mechanism participates in genome stability by affecting DNA repair, cell-cycle checkpoints, heterochromatin formation, and centrosome amplification. *BAP1* acts as a tumor suppressor gene and mutations disrupting its deubiquitinase activity or its nuclear localization abolish this function and drive cancer development [31]. Indeed, germline mutations in *BAP1* have been shown to be associated with an increased risk of developing MPM as well as other types of cancer, primarily melanoma. Furthermore, deep mutational profiling showed that *BAP1* is inactivated by either copy number or point mutations in about 57% of MPM cases, representing the most frequent alteration in this type of cancer. *BAP1* mutations seem to be associated to the epithelioid phenotype but not to a different prognosis [11]. Besides its function in DNA repair and genomic stability, *BAP1* also affects gene expression by altering histones’ post-translational modifications and chromatin accessibility. Furthermore, a direct activity of *BAP1* in defining stability of many transcription factors has been reported. Gene expression profiling of *BAP1* wild-type (wt) tumors showed profound changes in the transcriptional program of MPM cells. One thousand three hundred twenty-four genes were found differentially expressed in *BAP1* mutated tumors, including several HOXA genes as previously noted in experimental models [32]. In addition, among many TFs, YY1 had significantly reduced activity in *BAP1*-defective MPM cells. *BAP1* is known to form a complex with YY1 and HCF1 that represses transcription of genes involved in cell proliferation [33].

*NF2*, *TP53*, *LATS2*, *SETD2*, and to a lesser extent *SETDB1* are also found as frequently mutated in MPM. All five genes have high rates of nonsense, frameshift, and splice-site mutations causing their inactivation, in line with their functions as tumor suppressors. *SETD2* and *F* are both histone lysine methyltransferases. As *BAP1* alters the post-translational state of histones, these proteins control chromatin functions and the dynamics of many crucial processes, including DNA repair and gene expression.

*SETD2* is responsible for trimethylation of histone H3K36, which is found in gene coding regions and is associated with transcriptional elongation, peaking at the 3′ends of genes [34].

*SETD2*-dependent H3K36 trimethylation facilitates several processes within the cell, including splicing, repression of intragenic transcripts, and chromatin accessibility. *SETD2* is required in human cells for homologous recombination repair and in genome stability [35]. In fact, SETD2-deficient cancers exhibit a wide range of mutations, including insertions, deletions (indels), and chromosomal aberrations [36,37].

SET domain bifurcated histone lysine methyltransferase 1 (*SETDB1*) catalyzes histone 3 lysine 9 methylation, generating H3K9me2 and H3K9me3 histone marks. These modifications, preferentially associated to gene expression silencing, have been reported to hit on several pivotal genes involved in normal cell functions, such as TP53. Recent studies reported the overexpression of *SETDB1* in most cancer types, where it promotes cell proliferation, migration, and invasion [38]. Conversely, in MPM a significant percentage of loss of function mutations were detected in *SETDB1*, suggesting a different, not-yet-fully elucidated role of this gene in this type of tumor [39].

The neurofibromatosis 2 (*NF2*) gene is inactivated in 40–50% of MPM cases [40,41,42,43]. *NF2* is a tumor suppressor gene that encodes the moesin-ezrin-radixin-like (Merlin) protein that is associated with the actin cytoskeleton [42,43,44].

In its dephosphorylated form, Merlin accumulates in the nucleus where it inhibits the pro-oncogenic function of the E3 ubiquitin ligase CRL4 (DCAF1), thus conditioning the expression of several oncogenes [44,45]. In addition, Merlin inactivation leads to mTOR upregulation, promoting cell proliferation [46].

Moreover, Merlin loss, together with the inactivation of *LATS2* in a small number of patients, is believed to contribute to tumorigenesis through inactivation of the Hippo pathway [47]. As with Merlin, *LATS2* is also subject to point mutations and/or large deletions in about 11% of patients [48]. *LATS2*’s loss of function mutations correlates with bad prognosis [11,49,50].

Miyanaga et al. found alterations in the Hippo pathway both at the DNA and protein levels in several MPM cell lines and patients [51]. These perturbations lead to Hippo pathway inactivation with the consequent activation of the oncogene YAP1, which promotes the transcription of several cancer-promoting genes [47]. Moreover, YAP1 is often amplified in mesothelioma. YAP1 silencing by RNAi causes growth inhibition of MPM cells [51].

Bueno et al. found TP53 to be mutated in 8% of cases, a number higher than previously reported [8], but lower than in studies involving a smaller sample size [40]. However, TP53 is rarely mutated compared to other cancer types. TP53 mutations were absent from the epithelioid subtype. Moreover, patients with TP53 mutations showed lower overall survival than those with wild-type TP53, indicating the aggressiveness of TP53-mutant MPMs [8]. While all these studies provide precious details on the molecular basis of MPM, the wide genetic complexity and heterogeneity that emerge as specific features of this disease make it very difficult to translate this information in tools to improve patient management. In this regard, the use of algorithms or mathematical models integrating different types of information together with the genetic profile of the lesion will likely provide a better chance to assess the applicability of genetic analysis to resolve stratification of MPM patients.

## 4. Morphological Differentiation in MPM Is a Matter of Altered Gene Expression

In spite of extensive profiling, the most efficient MPM classification is based on morphology. According to a histological evaluation of cell differentiation, MPMs are subdivided in three main categories, epithelioid, sarcomatoid, and biphasic, the latest being a mixture of the other subtypes. Morphological grading also offers the best measure of risk stratification of these tumors, being that sarcomatoid the most aggressive and deadly form of MPM. Even if some attempts have been performed [52,53], genetic alterations seem not to account for this diversity. By contrast, hierarchical clustering on the basis of deep gene expression profiling always recapitulates these three categories, with biphasic MPM always in a wide and largely heterogeneous spectrum in between the other two histotypes. Recently, Blum and colleagues [54,55] have proposed that distinct morphological phenotypes correspond to distinct transcriptomic programs, thus MPM should be considered as a molecular gradient in between the two extreme differentiation phenotypes. Coupling deep transcriptional and epigenetic profiles with a deconvolution approach, these authors proposed that each MPM is a combination of a molecular epithelioid-like and sarcomatoid-like components. Proportion of these components was a reflection of histology and was strongly correlated with prognosis. In agreement, Hmeljak and colleagues [11] using multi-omic data from the TCGA cohort, showed that classification based on RNA profiles (including both coding and non-coding RNAs) is a good surrogate for the evaluation of MPM heterogeneity and reflects, even if in a finer way, the histological classification. On the one hand, this evidence provides new information about how epigenetic rewiring contributes to intra- and intertumoral heterogeneity in MPM and how such activity conditions clinical behavior in MPM. On the other, it offers new and finer instruments for MPM risk stratification, paving the way to more precise and tailored treatment approaches.

## 5. Epigenetic Events in MPM

Epigenetic modifications significantly affect gene expression and regulation without altering the DNA sequence. Epigenetic mechanisms are involved in several cellular processes, including tumorigenesis [56]. They include DNA methylation, histone modifications and chromatin remodeling. Moreover, non-coding RNAs such as micro-RNAs (miRNAs) and long non-coding RNAs, act as epigenetic regulators. Epigenetic alterations are being extensively studied in recent years since they are emerging as potential tools for an improved diagnosis and prognosis of MPM.

### 5.1. DNA Methylation of Tumor Suppressor Genes

DNA methylation involves the addition of a methyl group to the fifth carbon of the cytosine base, forming 5-methyl-cytosine. This reaction is catalyzed by a family of enzymes called DNA methyltransferases (DNMTs). This modification usually affects cytosines within CpG islands in promoters and other regulatory regions, causing the compaction of the chromatin structure. Promoter methylation inhibits the binding of the transcriptional machinery, resulting in gene silencing [57]. Global hypomethylation, together with the hypermethylation of many tumor suppressor genes (TSGs), is a signature of most cancer types, including MPM [58].

There are several studies reporting hypermethylation of TSG promoter in MPM as a consequence of asbestos exposure [12,26,59,60,61,62,63,64]. Christensen et al. compared the DNA methylation status of 803 cancer-associated genes in 158 mesothelioma specimens to 18 normal pleura samples [26]. They found a different methylation profile between tumor and normal tissue, and an association between higher methylation status and shorter overall survival. Moreover, they found a positive correlation between the methylation status and asbestos exposure. The genes that showed a different methylation state belonged to epigenetic regulation, cell cycle control, inflammation, and other pathways [26]. The same group also reported promoter hypermethylation, caused by asbestos exposure, in six cell cycle related genes (*APC*, *CCND2*, *CDKN2A*, *CDKN2B*, *HPPBP1*, and *RASSF1*), likely leading to an uncontrolled proliferation [12].

Goto et al. compared the methylation profiles of asbestos-associated MPM and lung adenocarcinoma samples [13], showing a common track of epigenetic modifications reaching 70% of deregulated genes. *TMEM30B*, *KAZALD1*, and *MAPK13* were selectively hypermethylated in MPM and their degree of methylation affected patients’ prognoses. In agreement with the negative prognostic value of DNA epigenetic modifications, hypermethylation was shown to be associated with advanced-stage and sarcomatoid phenotype [13].

Among TSGs epigenetically silenced in MPMs, many members of the secreted frizzled-related proteins (SFRPs), known as inhibitors of the Wnt pathway, have been reported [65]. Intriguingly, methylation in the promoters of these genes could be detected in patients’ plasma samples even if the small sample size investigated precluded a definitive conclusion. [61,66].

ZIC1 encodes for a family of zinc finger transcription factors involved in apoptosis. It acts as a tumor suppressor by inhibiting miR-23a and miR-27a, the overexpression of which is correlated to a shorter survival of MPM patients [67].

Interestingly, a differential distribution of methylation profiles was reported between the epithelioid and sarcomatoid subtypes. While in the sarcomatoid samples methylation changes occurred in particular within CpG sites, modifications in the epithelioid subtype were more widely distributed across the genome in non-CpG sites [54].

Besides affecting gene expression, methylated CpG sites are also prone to deamination, leading to missense mutations in cancer-related genes, thus contributing to the overall genomic instability [68].

It has also been reported that cytokines produced in response to the inflammation caused by asbestos exposure can dysregulate expression and/or targeting of methyltransferases (DNMTs) during the progression of MPM. Indeed, McLoughlin et al. found that the three major methylases are overexpressed in most of MPM cell lines [69]. The data from TCGA show that patients with overexpressions of DNMT1, DNMT3a, and DNMT3b have a higher level of methylation and, therefore, a shorter overall survival.

### 5.2. Histone Modifications and MPM

Histone modifications also affect gene expression, being responsible for the loosening and compacting of the chromatin structure. A causative association between histone modifications and MPM has not been fully established. Still, many of the genes known to be mutated in MPM (including *BAP1*, *SETD2*, and *SETDB1*) control histone post-translational features by primarily affecting their ubiquitination and methylation status. Furthermore, scattered evidence indicates a potential link between global chromatin acetylation and this disease. A decrease of acetylation of histones H3 and H4 is a common mechanism observed in various types of cancer and has been reported also in MPM [70,71]. Histone acetylation results in chromatin relaxation with consequent gene expression activation [72]. Lysine acetylation and deacetylation are catalyzed by histone acetyltransferases (HATs) and histone deacetylases (HDACs), respectively [73,74]. *HDAC1* and *HDAC2* are often dysregulated in many human malignancies, representing important therapeutic targets. Indeed, several HDAC inhibitors have been developed and show promising results for the treatment of many cancers, in particular hematological diseases. Sacco et al. found that *BAP1* modulates the expression of *HDAC2*, and indeed *BAP1* loss is linked to reduced expression of *HDAC2*, which in turn results in increased levels of *HDAC1*. In fact, a compensatory mechanism has been reported, so when *HDAC2* levels are low, *HDAC1* amounts increase. An increased sensitivity to HDAC inhibitors was observed following *HDAC2* or *BAP1* depletion, but not after *HDAC1* loss. Therefore, even if the total HDAC activity is maintained, it is likely that each isoenzyme has specific roles, and this finding suggests that specific inhibitors for *HDAC2* could drive more precise targeted therapy. The study opens the door for further investigations on sensitivity to HDAC inhibitors in patients with *BAP1* loss [75].

### 5.3. Micro RNAs

Non-coding RNAs are emerging as new key players in genomic function organization [76]. Micro RNAs (miRNAs) are short RNA molecules 18–25 nucleotides in length. Micro RNAs play different roles in several biological processes, both physiological and pathological. They exploit their function at a post-transcriptional level by binding to target mRNAs in a sequence-specific manner. A single miRNA can regulate several mRNAs and each mRNA can be regulated by different miRNAs. Micro RNA binding to its target mRNA results in its degradation, inhibiting its translation [77,78]. Micro RNAs are already used as diagnostic tools for several diseases, and they can be found both in tissues and in biological fluids [79,80]. Many studies were carried out to explore the specific miRNA expression profile of MPM. It was reported that asbestos causes overexpression of miR-374a, miR-24-1, let-7d, let-7e, miR-119b-5p, miR-331-3p, and miR-96 and downregulation of miR-939, miR-671-5p, miR-605, miR-1224-5p, and miR-202 [81,82]. Reid et al. found a downregulation of the miRNA 15/16 family in MPM tumors compared to normal mesothelial tissue. Moreover, overexpression of miR-16 inhibited proliferation of MPM cells [83]. Additionally, miR-16 acts as a tumor suppressor gene and its expression was correlated to patients’ survival [84,85]. Downregulation of miR-16, together with downregulation of miR-15b, miR-195, and miR-200c, is associated with an increased expression of programmed death-ligand 1 (PDL1) [28].

Another tumor suppressor gene that is often downregulated in MPM patients is miR-17 [85].

Downregulation of miR-126 is associated with upregulation of VEGF, resulting in increased vascularization and enhanced metastasis. Several studies observed a downregulation of this miRNA in MPM patients’ plasma [85,86,87], but the discrimination between MPM patients and controls was not very high.

## 6. Epigenetic Features as Diagnostic and Prognostic Markers in MPM

While the etiology of MPM is becoming clearer due to recent genomic studies, poor advances have been made in the daily management of the disease. MPM is usually diagnosed in advanced stages, when treatments and procedures are very limited and not very effective. Thus, tools that may improve diagnosis and prognosis of MPM patients are currently an urgent clinical need.

Some of the epigenetic modifications reported above can be detected in biological fluids, such as plasma, serum, urine, or cerebrospinal fluid. These molecules, reported as “circulating biomarkers”, are receiving particular attention with regard to cancer diagnosis for their non-invasive collection method [88]. Moreover, epigenetic alterations have been often found in early-stage patients [24,69], thus representing a promising tool for an early diagnosis.

Very few studies have focused on alterations of DNA methylation in blood as circulating markers of MPM, and, in spite of the promising results, the seemingly limitless results gathered from this type of analysis are so far a major issue in the translational applicability of this information. Recently, DNA methylation status was investigated in the peripheral blood of 163 MPM patients and compared to 137 healthy controls. The analysis revealed more than 800 differentially methylated CpG sites in the MPM cohort. The three major hypomethylated CpG sites corresponded to FOXK1, MYB, and TAF4, while the most hypermethylated CpG sites were *CXCR6*/*FYCO1*, *TAP1*, *MORC2*, and *LIME1*. These differentially methylated CpG sites showed a diagnostic value, and the results were stable across the different MPM histotypes [89].

In a separate study, Fischer et al. used two-stage methylation-specific PCRs to study the methylation status of nine promoters in the serum DNA of 43 patients with malignant mesothelioma, showing that the combined hypermethylation of RARβ, DAPK, and RASSF1A promoters, known for their tumor-suppressor activities, was associated with shorter overall survival [90]. While promising, some considerations must be undertaken in evaluating the transferability of these applications in real life. First, circulating DNA levels are widely influenced by dimension, state, and diffusion of the tumor. Moreover, DNA methylation detected in blood only partially reflects the methylation state of the tumor component, being largely representative of the activation of immune response toward cancer cells and/or other systemic biological processes. Surely, the use of larger and homogenous patients’ cohorts is needed in order to define the real potential of these applications as diagnostic or prognostic biomarkers in MPM.

Over the past decade, several studies have been conducted to explore circulating miRNAs in MPM (reviewed in [88,91]). Santarelli et al. reported a downregulation of miR-126 in serum samples of MPM patients compared to either asbestos-exposed people or unexposed healthy controls. However, the sensitivity and specificity of this marker was modest. Additionally, miR-103 was downregulated in the blood of 23 MPM patients compared either to 17 people exposed to asbestos or 25 healthy controls. The discrimination between these groups based on miR-103 expression was higher than on miR-126 expression [92].

These data were confirmed by Tomasetti et al., who, in a cohort of 45 MPM patients and 56 healthy controls, showed that miR-126-3p discriminates MPM patients with a sensitivity of 80% and a specificity of 60% [87]. Noticeably, the diagnostic performance of miR-126-3p seemed significantly improved when combined with the analysis of mesothelin and methylation of the thrombomodulin promoter (AUC 0.857, 95% CI 0.767–0.927) [93].

For the first time, Cavalleri et al. explored the miRNA expression profile in extracellular vesicles, finding a differential expression between MPM patients (n = 23) and asbestos-exposed healthy subjects (n = 19). They identified the combination of miR-103a-3p and miR-30e-3p as the most discriminating one, generating an AUC of 0.942 (95% CI 0.87–1.00) with a sensitivity of 95.5% and a specificity of 80% [94]. As well, Kirschner et al. showed that higher serum levels of miR-625-3p discriminate MPM patients from asbestos-exposed healthy subjects with an accuracy of 79.3%, sensitivity of 70%, and specificity of 90% [95].

Serum analysis of miR-548a-3p and miR-20a levels in 60 MPM patients revealed overexpression of both in comparison with 20 asbestos-exposed people and 20 healthy subjects. The combination of the two miRNAs reached a sensitivity of 100% [96]. A recent study showed that high levels of the long non-coding RNA RP1-86D1.3 and miR-2053, together with low levels of damage-regulated autophagy modulator (DRAM1) and arylsulfatase A (ARSA) mRNAs, were associated with MPM and could efficiently discriminate MPM (n = 100) from exposed or not healthy subjects. Moreover, the authors suggest that the upregulation of miR-2053 could also be a good prognostic marker of MPM [97].

Lamberti et al. compared miRNA expression in the serum of 14 MPM patients to 10 controls and observed upregulation in five miRNAs (miR-101, miR-25, miR-26b, miR-335, and miR-433) and downregulation in two miRNAs (miR-191 and miR-223). Based on these results, they proposed two miRNA signatures using different combinations of up- and downregulated miRNAs for histotype and survival predictions [98].

Considering together all these studies, the possibility of considering circulating miRNAs as potential diagnostic and/or prognostic biomarkers for MPM has been attempted for almost ten years without reaching a definitive consensus.

This is in part attributable to technical limitations like different methodologies, choice of normalization methods, and types of controls. Additionally, a major drawback of these studies is the fact that they were performed on cross-sectional cohorts and focused on patients with late-stage diseases, leaving unexplored the performance of miRNAs as early diagnostic markers.

Indeed, when tested in this context, as recently demonstrated by Weber and colleagues, who investigated miR-132-3p, miR-126-3p, and miR-103a-3p in prediagnostic plasma samples, miRNAs fail to detect cancer, showing their limitation as early detection markers for malignant mesothelioma [99].

## 7. Epigenetics and Microenvironments a Dangerous Crosstalk in MPM

Clinical management of MPM is highly challenging. Part of this challenge is due to the intimate connection of MPM cells with the surrounding microenvironment.

Several studies have highlighted the complex role that both local and systemic inflammation plays in the development and progression of many types of cancer, including MPM [100]. The activation of local immune response seems to be correlated in MPM with an aggressive disease and worse prognosis [101,102]). A very heterogenous immune infiltrate has been described in MPM with a predominant role for tumor-associated macrophages (TAM) and tumor-infiltrating CD4+ and CD8+ T lymphocytes. Different frequencies of these cells in the tumor milieu seems to hold specific prognostic value. Together with additional infiltrating stromal cells like cancer-associated fibroblasts (CAFs), these immune populations secrete pro-inflammatory signals that create a very reactive local environment. Many of these signals, including TGFb or pro-inflammatory cytokines, are known to trigger intracellular downstream signals that converge on changes in the gene expression program of cancer cells by affecting the epigenomic landscape of these cells (reviewed in [103]).

Indeed, consistent evidence demonstrates that chromatin rewiring in cancer cells is heavily conditioned by the microenvironment and, in turn, changes in the epigenome of the tumor may alter features of the neighboring cells. The immune system plays a fundamental role in this regard. We have already mentioned how the massive inflammatory state of the microenvironment promotes MPM development and progression, hitting, among others, on the epigenome of cancer cells.

Chronic inflammation of the pleura caused by asbestos exposure during MPM development triggers apoptosis or necrotic death of mesothelial cells, inducing the release of alarmins and the accumulation of nucleosides and nucleotides in the extracellular pleura space. These molecules are able to induce an immunosuppressive effect and accelerate metastasis, influencing the signaling necessary for macrophages and cytotoxic T-lymphocytes activation [104]. Sigalotti et al. [105] showed for the first time that the cancer testis antigens (CTAs), which are completely absent in healthy tissues, are highly expressed, even if with heterogeneous levels, in MPM cells. Expression of CTAs is usually linked to cancer progression and negative immunomodulation, being able to be recognized by macrophages and dendritic cells and to block their antitumor activity. CTA expression is known to be primarily regulated by epigenetic events. In particular, both DNA methylation and histone modification silence these genes under normal conditions.

At the same time, various HDAC inhibitors, such as sodium butyrate, SAHA, and valproic acid, have been demonstrated to have important anti-inflammatory properties, affecting the expression of pro-inflammatory cytokines and promoting the differentiation of naïve CD4+ T lymphocytes into regulatory T cells (Tregs) with increased immunosuppressive activity [104].

## 8. Targeting Epigenome: New Strategies for Potential Therapies

MPM is usually diagnosed in advanced stages when surgical treatments are often limited. Surgery, chemotherapy, and radiotherapy have so far been the only therapeutic options. Recently, the open-label phase III CheckMate 743 clinical trials showed that administration of ipilimumab plus nivolumab results in a significant improvement of patients’ survival (median OS 18.1) as compared to standard chemotherapy, thus providing the basis for the introduction of immunotherapy as an additional therapeutic option for MPM patients [106]. In spite of this, these strategies often reach only modest therapeutic effects, leaving MPM patients with poor life expectancy [107], suggesting the urgent need for new treatment approaches for this tumor. In parallel, the development of MPM-oriented target therapies has been so far limited by both lack of activating driver mutations and the limited knowledge about the molecular basis of this tumor. The collective amount of evidence pointing to epigenetic alterations as important determinants in MPM development and prognosis lays instead the groundwork for numerous preclinical and clinical studies using epigenetic drugs to target MPM (Table 1).

Indeed, inactivation of tumor suppressor genes, driven by promoter hypermethylation [58] and chromatin hypoacetylation [70], is a distinctive feature of MPM, paving the way for the use of DNMT inhibitors (DNMTis) and HDAC inhibitors (HDACis) in several preclinical studies alone or in combination with traditional chemotherapy.

In 2009, in vitro studies showed that the use of the HDACi valproic acid coupled with cisplatin and pemetrexed in MPM cell lines was effective in inducing caspase-dependent apoptosis, and the same treatment performed in mouse xenograft models showed complete suppression of MPM growth [108,109]. Successively, a phase II study tested a combination of valproic acid with doxorubicine in second-line therapy of MPM patients, observing a partial response in 6/45 patients with a good performance status [110].

In line with these data, Hurwitz et al. demonstrated the overexpression of FLICE-inhibitory protein (FLIP) and procaspase 8 in MPM patients and the efficacy of the HDACi SAHA (vorinostat) in inducing FLIP downregulation and Caspase 8 activation with a consequent apoptotic response in MPM cells [111]. Moreover, a combination of vorinostat with cisplatin in MPM cell lines resulted in higher induction of apoptosis when compared with either agent alone [111]. However, in 2015 a phase III randomized, placebo-controlled trial comparing vorinostat versus a placebo in second- and third-line therapy of MPM patients observed no significant improvement in overall survival [112].

The lack of effect of this single drug in MPM cannot be considered totally unexpected and was suggested in several preclinical studies regarding its combination with DNA demethylating agents.

It was demonstrated that the 5-aza-2′deoxycytidine (decitabine), a DNA methyltransferase inhibitor, is able to increase the expression of genes involved in DDR and cell cycle regulation, inducing a growth arrest of MPM cells typical to senescence [113].

Additionally, decitabine alone or in combination with two different HDACis (valproic acid and SAHA) was used to treat MPM cells. Results showed a synergic effect between these two distinct classes of drugs in inducing tumor-associated antigen expression and consequent activation of cytotoxic T cell response. Moreover, the sequential treatment of murine models of mesothelioma with decitabine and valproic acid was demonstrated to significantly reduce tumor progression [114]. The same researchers, in a subsequent paper, obtained similar results testing new more potent and convenient HDACis in combination with decitabine. Besides confirming the cytotoxicity of these drugs, these authors also supported the rational for the combination of epigenetic drugs with an anti-PD-L1 targeting strategy [115] and further highlighted the tight connection existing between epigenetic re-wiring and the cancer microenvironment.

Since the discovery of the role of super-ENHs and of the importance of BRD4 and BET proteins in their activation [116], BET-targeting agents (BETis) were regarded as promising drugs in cancer. In 2018, Riganti et al. [117] tested the efficacy of BETis in MPM cells, demonstrating the capability of these drugs to induce an immunogenic response against MPM cells and showing that JQ1 treatment restrains tumor growth in immunocompetent MPM mouse models. Being better tolerated than methyltransferase inhibitors and HDACis [118], these drugs may become a more effective way to target alone or in combination the epigenome of MPM.

In spite of promising preclinical data and a solid rationale, clinical trials testing epigenetic drugs’ efficacy in MPM are rare, likely due to the high toxicity of these compounds and their short half-life and poor body distribution that limited their use in patients with solid tumors [69].

Still, the mechanicistic synergy that these drugs have with other anticancer drugs suggests that the accurate design of combinatory trials for converging on specific biological processes would be a potential strategy for the employment of these drugs in the management of many solid cancers that, like MPM, have very few targeted therapies available [119,120]. In this regard, the recent indication of immune therapy efficacy in treating MPM opens a new and still partially explored route. Indeed, all classes of epigenetic drugs have been shown to modulate immune response acting either on immune cells infiltrating the tumors or in modulating the crosstalk between cancer and immune cells via the modulation of specific mediators. Several ongoing trials are testing combinations of HDACis or BETis with immunotherapic agents in several settings and providing encouraging preliminary evidence [121].

**Table 1:** Epigenetic drugs tested in clinical and preclinical trials for MPM treatment.

Drug	In Combination With	Effect	Trial	Reference
Valproic acid (HDACi)	Cisplatin and pemetrexed	Increased apoptosis of MPM cells; suppression of MPM growth in mouse xenograft models	Preclinical	Vandermeers, F. et al., 2009 [108]
Valproic acid (HDACi)	Doxorubicin	Partial response in 6 of 45 second-line MPM patients	Phase II clinical trial	Scherpereel, A. et al., 2011 [110]
SAHA (HDACi)	-	Caspase 8 induced apoptosis	Preclinical	Hurwitz, J.L et al., 2012 [111]
SAHA (HDACi)	Cisplatin	Increased apoptosis compared to the single drugs		
SAHA (HDACi)	-	No increase in patients’ OS compared to placebo	Phase III clinical trial	Krug, L.M. et al., 2015 [112]
Decitabine (DNMTi)	-	Induction of genes involved in DDR and cell cycle regulation, causing premature senescence induction	Preclinical	Amatori, S et al., 2011 [113]
Decitabine (DNMTi)	Valproic acid/ SAHA	Induction of tumor-associated antigens and activation of cytotoxic T cell response; reduction of tumor progression in mice	Preclinical	Leclercq, S. et al., 2011 [114]
Decitabine (DNMTi)	Newly synthetized HDACi	Increase in PD-L1 expression	Preclinical	Bensaid, D. et al., 2018 [115]
JQ1 (BETi)	-	Induction of immunogenic response; inhibition of tumor growth in immunocompetent mice	Preclinical	Riganti C. et al., 2018 [117]

## 9. Conclusions

MPM is still one of the most aggressive and deadly forms of cancer. Its silent evolution, the generally old age of the patients, and the lack of effective therapeutic strategies make MPM clinically hard to manage and leaves MPM patients with a very poor prognosis. Away from being an “old fashioned” disease, MPM patients require the elaboration of new strategies to improve both life expectancy and quality of life. The epigenome is the control center of gene expression. Working as a rheostat, the epigenome modulates the expression of crucial genes, thus facilitating the acquisition of essential features for cancer survival. This seems to be extremely important in MPM, which is recognized as a “loss of function” type of cancer characterized by alterations leading to inactivation of onco-suppressive genes. Epigenetic modifications increasing the structural complexity of specific loci are used to turn off the expression of such genes driving MPM progression. Additionally, epigenetic alterations in MPM may affect the way cancer cells are recognized by the immune system, leading to immune evasion and resistance to immune checkpoint inhibitors. While single-agent epigenetic drugs have shown poor applicability in treating solid cancers, used in combination with chemotherapy or other genome targeting drugs, they may represent a new therapeutic perspective regarding this tumor. Furthermore, looking at the dark side of the genome, including the exploration of non-coding RNAs, may help to shed light onto the molecular basis of this cancer, providing answers that so far are still missing.
